# Glottic-SubGlottic adenoid cystic carcinoma. A case report and review of the literature

**DOI:** 10.1186/1471-2482-13-S2-S48

**Published:** 2013-10-08

**Authors:** Domenico Testa, Germano Guerra, Giovanni Conzo, Michele Nunziata, Gioacchino D'Errico, Maria Siano, Gennaro Ilardi, Mario Vitale, Francesco Riccitiello, Gaetano Motta

**Affiliations:** 1 Department of Otolaryngology, Head and Neck Surgery, Second University of Naples, Naples, Italy; 2 Department of Medicine and Health Sciences, University of Molise, Campobasso, Italy; 3 Department of Anesthesiology, Surgery and Emergency, Second University of Naples, Naples, Italy; 4 Department of Advanced Biomedical Science, Unit of Pathology, University of Naples "Federico II", Naples, Italy; 5 Department of Medicine and Surgery, University of Salerno, Baronissi (SA), Italy; 6 Department of Neuroscience and Reproductive and Dentistry Sciences, University of Naples "Federico II", Naples, Italy

## Abstract

**Background:**

Malignant tumours of minor salivary glands are uncommon, representing only 2-4% of all head and neck cancers. In the larynx, minor salivary gland tumours rarely occur and constitute less than 1% of laryngeal neoplasm. Most of the minor salivary gland tumours arise in the subglottis; however, they can also occur in the supraglottis, in the false vocal cords, aryepiglottic folds and caudal portion of the epiglottis. The most common type of malignant minor salivary gland tumour is adenoid cystic carcinoma.

**Methods:**

We present a unusual case of adenoid cystic carcinoma of glottic-subglottic region in a 61-year-old woman. Follow-up endoscopy and laryngeal magnetic resonance imaging (MRI) at three years after treatment showed no recurrence of the tumour.

**Results:**

The diagnosis of glottic-subglottic adenoid cystic carcinoma should be considered in patients who are characterized by dyspnea, cough and stridor, but do not respond to pharmacologic approach.

**Conclusions:**

Adenoid cystic carcinoma is usually a very slow growing cancer, invested by an apparently normal laryngeal mucosa, so that it can show no clear symptoms for a long time. For these reasons the increasing number of diagnostic mistakes or late diagnosis that may be fatal in some cases.

## Introduction

Adenoid cystic carcinoma is the predominant histologic type among malignancies of the minor salivary glands, with a frequency of 10-20%, representing only 2-4% of all head and neck malignancies [1,2]. In the population the higher incidence is characteristic for women, especially between 50/60 years. There are no distinct risk factors that predispose patients to this malignancy. Smoking does not affect the incidence [3,4]. Its predominant site is in the salivary glands of the oral cavity, in particular in the hard palate and whit lesser frequency in the nasal cavity, paranasal sinuses, pharynx and larynx, in this last localization is incredibly rare (0.07 %- 0.25% of all laryngeal tumours, 1% of all ACC), there are a few salivary glands in the mucosa of laryngeal-tracheal tract [2,3,5]. Only 174 cases of ACC in the larynx have been reported in literature reviews, with a percentage of incidence, in the anatomic laryngeal subsites as follows : supra-glottis region (25%), glottis region ( 5%), transglottic area ( 6%), sub-glottis region ( 64%). The local diffusion is frequently infiltrating, with a slow evolution and a large local invasiveness (thyroid/esophagus); it's possible, anyway, the exophytic or polipoid growth of cancer in particular in the laryngeal-tracheal site [6]. The dyspnea is often the predominant symptom and the local-regional metastases, at least for malignancies originated from subglottis region, are almost rare. The average survival is about eight years. The evolution prognosis of this disease is caused by lungs, bones and brain metastases or by local recurrence, so thanks to slow tumour growth, it sometimes could recur after ten or more years [7]. We report a case of ACC, treated as nodular benign thyroid neoplasm, in a patient whit previous diagnosis of asthma.

## Case report

**Figure 1:**
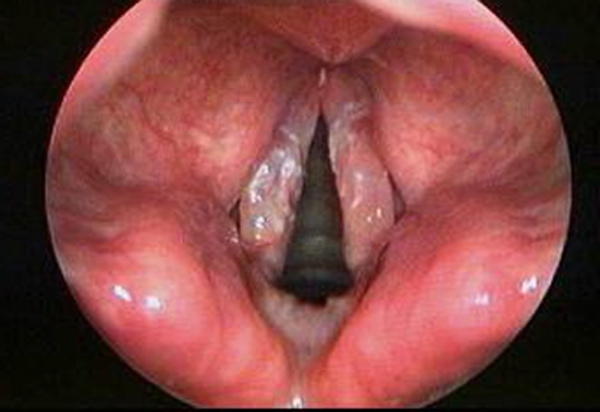
**Fiberlaryngoscopic finding that show a ACC of left vocal fold**.

**Figure 2:**
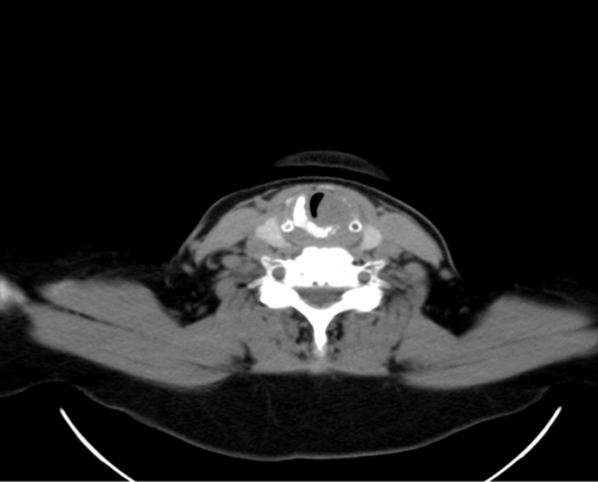
**CT scan of larynx showing left vocal cord mass and destruction at left side of thyroid cartilage**.

**Figure 3:**
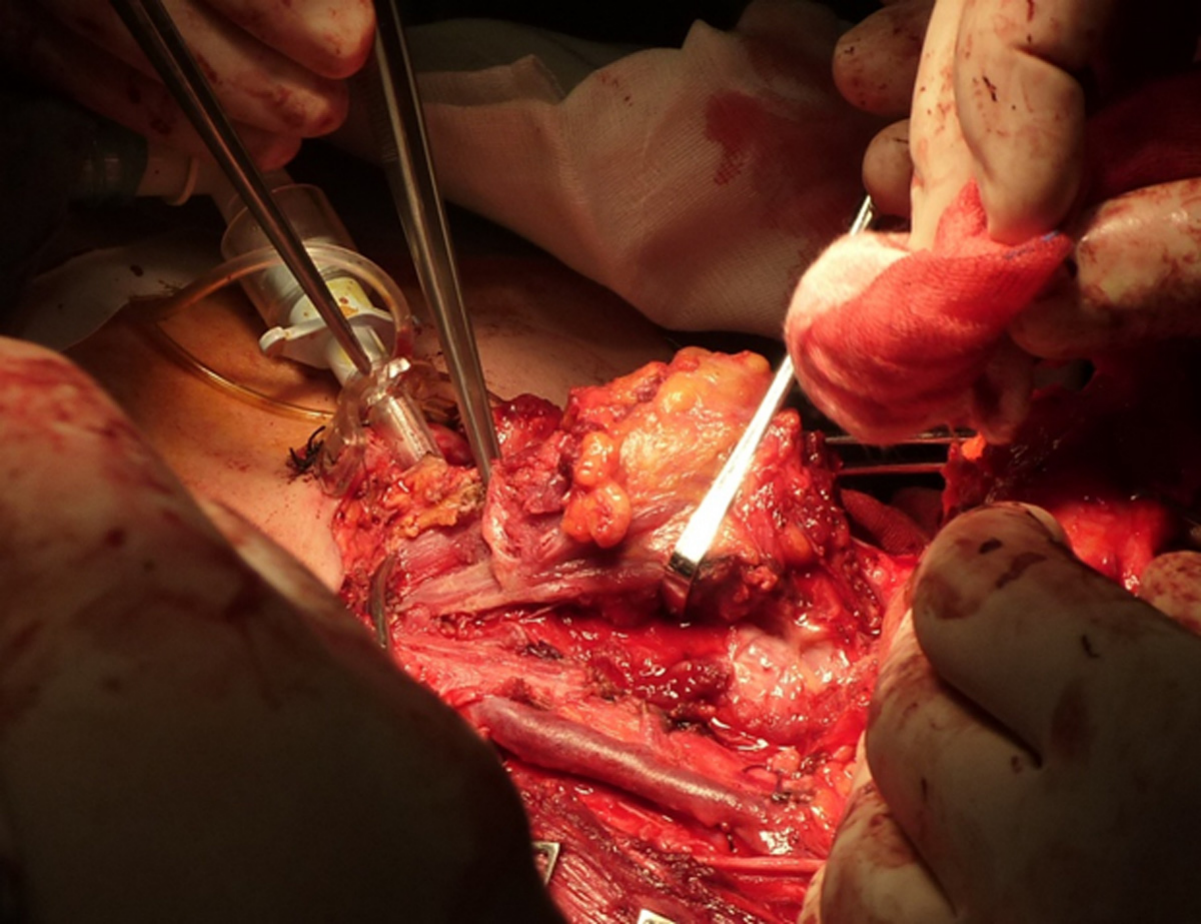
**Total laryngectomy with functional bilateral lymph node excision of II-III-IV-V-VI level**.

**Figure 4:**
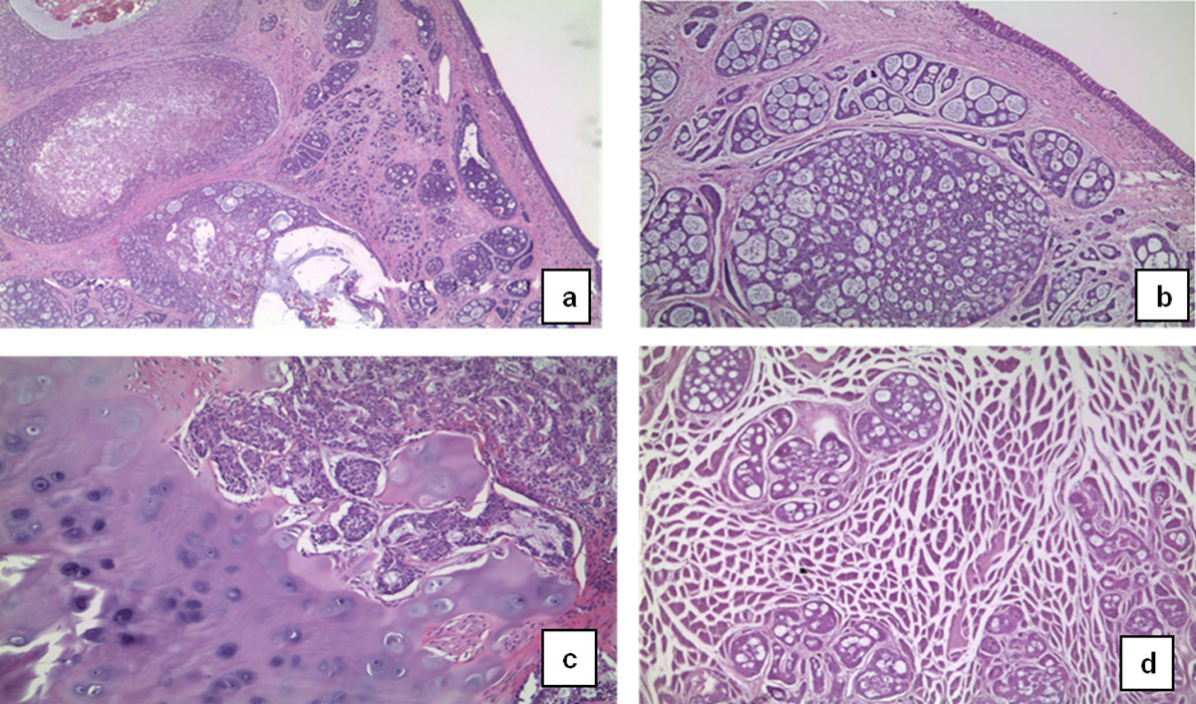
**The figures show adenoid cystic carcinoma deeply infiltrating the laryngeal mucosa**. The tumour shows a predominant cribiform pattern, and is composed of pseudocysts, filled with basophilic mucin, surrounded by basaloid cells **a, b**.. Areas of intra-tumoural necrosis are also present. The lesion involves the cartilage (**c**) and the striated muscle (**d**) (Hematoxylin - eosin; a: 50X; b: 100x; c:200x; d:200x)

A.N, 61 year-old woman, with an ACC injury in the glottis-subglottis region, has been examined after a surgical treatment of thyroid. The clinical appearance of thyroid cancer is that of a nodules, some time representing a challenging diagnostic dilemma with thyroid or unusual extrathyroidal masses [8,9]. The patient, suffering from severe dyspnea, was seen by the general surgeon at first, because she was affected by a thyroid disease, causing compression, dislocation and reduction of tracheal caliber. The ultrasound exam of thyroid confirmed a nodularity and the functionality exams showed an evident dysfunction of gland. The FNAC, made in hospital, didn't show diagnostic significance. The fiberlaryngoscopy control, effected before thyroidectomy, reported a moderate reduction of hypoglottic space, associated with a vocal cord hypomobility, especially on the left. The persistence of dyspnea, after the thyroidectomy, and the final pathological examination characteristic for ACC, made us doubt about the origin of lesion in that site. The patient arrived to our observation for a further diagnostic analysis. In fact, the fiberopticlaryngoscopy showed an hypertrophy of a left vocal fold, in the midline position, with a slightly uneven surface, ipsilateral hypoglottic region and subcommissurale region neoformation and narrowing of the lumen (Figure 1). The subsequent cervicothoracic CT shows a nodular lesion of left part of larynx, associated with partial destruction of thyroid cartilage, cricoid ring to the left and ipsilateral arytenoid cartilage, in presence of bilateral cervical lymph node hyperplasy (Figure 2). According to the clinical and radiologic data, the patient was submitted to a total laryngectomy with functional bilateral lymph node excision of II-III-IV-V-VI level (Figure 3). The pathological examination confirmed a adenoid cystic carcinoma deeply infiltrating the laryngeal mucosa, cricoid carticalgine and striated muscle tissue present, with a predominantly cribiform pattern, consisting of pseudocytos, filled with mucin basophilic basaliod cells, surrounded by areas of intra-tumoural necrosis and absence of cervical lymphnode metastases (Figure 4). According to the histological evaluation and to neoplastic involvement (T3N0), the patient underwent to radioterapy on "T" and "N", with a total dose of 66GY, subdivision in 200cGy/die. The clinical and radio follow up after 6, 12, 24, and 36 months, consisting of ultrasound, TC, PET total body, showed the absence of recurrence. No images are shown of the primitive residue, referring to glottis - subglottic ACC.

## Discussion

Aging is accompanied by a increased incidence of mortality from many diseases like cancer, diabetes, neurodegenerative, cardiovascular and other pathologies all related to oxidative stress and elevated ROS (Reactive oxygen species) [10,11]. Drugs, chemicals, pollutants, high-caloric diets, exercise and sport activity are stress agents involved in overproduction of oxidant molecules[12]. The most common malignancy of the larynx is squamous cell carcinoma. Median age of occurrence is in the sixth and seventh decades and less than 1% of laryngeal cancers arise in patients younger than 30 years of age. Less commonly, other epithelial, neuroendocrine, and soft tissue tumours can also occur in this location [13,14]. Laryngeal salivary gland carcinomas are rare diseases, cause the low density of minor glands in the larynx (between 23 and 47 glands/cm2) accounting for less than 1% of all laryngeal malignancies, the most common type is adenoid cystic carcinoma [15,16]. No distinct risk factor that predisposes patients towards this malignancy has been identified [3]. Smoking does not affect the incidence differently from what happens for squamous cell carcinoma [4]. According to Dexemble et al. (2003) laryngeal ACC arises in sub-glottis area, which could be an isolated site of neoplasm (60%) or could be invaded by glottis or supra-glottis neoplasm (20%) [6,15]. Neck metastasis are rare (10 to 15% of the cases), but distant recurrence is common mainly to the lung and may occur many years after the primary neoplasm surgical treatment [3,16]. Adenoid cystic carcinoma is usually a very slow growing cancer, invested by an apparently normal laryngeal mucosa, so that it can show no clear symptoms for a long time. For these reasons the increasing number of diagnostic mistakes or late diagnosis that may be fatal in some cases [4]. The symptomatic square such as dyspnea, dysphagia and dysphonia becomes clear in the respective supraglottic, glottic, hypoglottic sites and it is the most insidious form above all, in case of a late diagnosis. The signs and symptoms of laryngeal adenoid cystic carcinoma are related to location and size. Tumours of the supra-glottis present with dysphagia. Hoarseness or even dyspnea is indicative of glottic involvement. Stridor and airway obstruction are more frequently associated with sub-glottic tumours. However, usually, adenoid cystic carcinoma occurs as a large asymptomatic, non-ulcerated sub-mucosal mass. As a result, diagnosis is often delayed and, in the larynx, sub-glottic tumours have the opportunity to invade deeply before they are diagnosed [2]. The slowly insurgent dyspnea together with laryngeal screeching may lead to an early diagnose of asthma and if the symptoms don't disappear despite the adeguate therapy, radiological finding such as CT is always most important for the diagnosis [5]. The direct invasion of the thyroid by the ACC caused by the laryngotracheal complex simulating a nodular benign pathology of the thyroid, just like the case we have discussed so far, is a rare occurrence that can delay the diagnosis [17]. The histopathological pattern of adenoid cystic carcinoma is classified into three distinct subtypes: cribriform, which is the most common form and characterize case described here, tubular, which has the best prognosis; and solid, which carries the worst prognosis [3]. The five-year survival rates for patients with laryngeal Adenoid Cystic Carcinoma have been reported in literature to range from only 12 to 17% after surgery [3,4]. Surgical excision is the main treatment due to the relative radioresistance of these tumours [2,18]. Total laryngectomy assures radical tumour excision and is often required because of submucosal spread, lymphatic diffusion and perineural invasion [2]. Postoperative radiation therapy is recommended, since radiotherapy has shown result in prolonging survival and preventing local recurrences [19]. The use of Standard chemotherapies have systemic toxicities and limited efficacy in the case of larynx carcinoma as well as of other more common solid tumours [20,22]. Some authors have reported positive responses to chemotherapy, recommending it as palliative therapy in advanced cases [23]. Conversely, there is no hint of a remodeling of the Ca^2+ ^toolkit, that has been observed in other malignancies, including renal cellular carcinoma [24,25] and prostate cancer [26], and has been put forward as alternative target for selective molecular therapies [23].

## Conclusion

Laryngeal salivary gland carcinomas are rare and account for < 1% of laryngeal malignancies. Therefore, a high degree of suspicion is essential for early diagnosis. This tumour must be considered when aggressive laryngeal tumours are found, especially if the patient is not at risk for squamous cell carcinomas. They usually originate in the supra-glottic or sub-glottic area with a predominance of old age. Most patients are diagnosed late, at an advanced stage. CT can be used to assess tumour extent and growth patterns. Wide-margin surgery alone or in combination with post-operative radiotherapy for advanced lesions that present peri-neural spread or close or positive margins is the best tumour management. For these patients, regular and long-term follow-up is mandatory, in order to detect relapses and metastases.

## Competing interests

The authors declare that they have no competing interests.

## Authors' contributions

DT: conceived the study, analyzed and interpreted the data, drafted the manuscript. GG: conceived the study, analyzed and interpreted the data, drafted the manuscript. GC: critically revised the manuscript. MN: critically revised the manuscript. GD: critically revised the manuscript. MS: analyzed the data. GI: analyzed the data. MV: critically revised the manuscript. FR: critically revised the manuscript. GM: conceived the study, critically revised the manuscript. All authors read and approved the final manuscript.

## Authors' information

DT: Assistant Professor of Otolaryngology at Second University of Naples

GG: Assistant Professor of Anatomy at University of Molise

GC: Assistant Professor of Surgery at Second University of Naples

MN: Resident in Otolaryngology Training Program at Second University of Naples

GD: Assistant of Otolaryngology at Second University of Naples

MS: Research Fellow at University of Naples "Federico II"

GI: PhD Student at University of Naples "Federico II"

MV: Assistant Professor of Endocrinology at University of Salerno

FR: Assistant Professor of Dentistry at University of Naples "Federico II"

GM: Full Professor of Otolaryngology at Second University of Naples

## Declarations

Publication charges for this article were covered by research funds of the project Bando Faro 2012 - Finanziamenti per l'Avvio di Ricerche Originali, cofounded by the Compagnia di San Paolo and by the Polo per le Scienze e le Tecnologie per la Vita of the University Federico II in Naples.

This article has been published as part of *BMC Surgery *Volume 13 Supplement 2, 2013: Proceedings from the 26th National Congress of the Italian Society of Geriatric Surgery. The full contents of the supplement are available online at http://www.biomedcentral.com/bmcsurg/supplements/13/S2
